# Using Deep Learning to Identify Costa Rican Native Tree Species From Wood Cut Images

**DOI:** 10.3389/fpls.2022.789227

**Published:** 2022-04-01

**Authors:** Geovanni Figueroa-Mata, Erick Mata-Montero, Juan Carlos Valverde-Otárola, Dagoberto Arias-Aguilar, Nelson Zamora-Villalobos

**Affiliations:** ^1^School of Mathematics, Costa Rica Institute of Technology, Cartago, Costa Rica; ^2^School of Computing, Costa Rica Institute of Technology, Cartago, Costa Rica; ^3^School of Forestry Engineering, Costa Rica Institute of Technology, Cartago, Costa Rica; ^4^Cooperativa de Productividad Forestal, Facultad de Ciencias Forestales, Universidad de Concepción, Concepción, Chile

**Keywords:** deep learning, convolutional neural network, plant classification, automated image-based tree species identification, costa rican tree species, xylotheques

## Abstract

Tree species identification is critical to support their conservation, sustainable management and, particularly, the fight against illegal logging. Therefore, it is very important to develop fast and accurate identification systems even for non-experts. In this research we have achieved three main results. First, we developed—from scratch and using new sample collecting and processing protocols—an dataset called *CRTreeCuts* that comprises macroscopic cross-section images of 147 native tree species from Costa Rica. Secondly, we implemented a CNN for automated tree species identification based on macroscopic images of cross-sections of wood. For this CNN we apply the fine-tuning technique with VGG16 as a base model, pre-trained with the ImageNet data set. This model is trained and tested with a subset of 75 species from CRTreeCuts. The top-1 and top-3 accuracies achieved in the testing phase are 70.5% and 80.3%, respectively. The Same-Specimen-Picture Bias (SSPB), which is known to erroneously increase accuracy, is absent in all experiments. Finally, the third result is Cocobolo, an Android mobile application that uses the developed CNN as back-end to identify Costa Rican tree species from images of cross-sections of wood.

## 1. Introduction

Costa Rica is one of the countries with more tree species in the world. Within its 51, 900 *km*^2^ it has around 2, 300 species distributed in approximately 700 genus and 130 families. These figures are even more significant when we compare them to those of other regions. For example, the continental United States of America has about 700 species over a territory of about 10 million square kilometers (Fournier Origgi, 2016).

Trees are essential to sustain life on Earth, particularly, human life. They provide raw material for production of many goods and services, protect watersheds and wildlife, improve air quality, and help counter climate change by removing carbon dioxide from the air, storing carbon, and releasing oxygen into the atmosphere, among many other environmental services. Costa Rica has carried out important conservation efforts, such as the PAS (Pago por Servicios Ambientales) program, which grants financial recognition to farm owners who establish reforestation projects or natural regeneration (ONF, 2020). It has also enacted laws that attempt to prevent illegal logging of tree species (SCIJ, 1996; Quesada-Monge, 2004). Thus, an accurate and fast tree species identification is vital for their conservation and proper management. Accordingly, tree species identification has become a challenge for environmental ministries and environmental organizations in all countries.

Because, the identification of tree species is also of great value in legal, commercial, industrial, forensic, and paleontological contexts, where only samples of wood are present, an identification based solely on wood samples is often needed.

**Figure 1:**
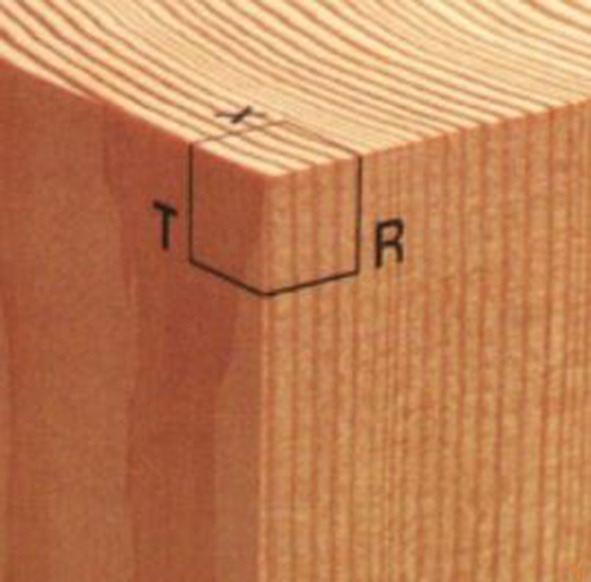
Cutting planes (taken from Hoadley, 2000).

Wood species identification can be performed at a microscopic and/or macroscopic level. The former is more accurate than the latter, but requires special equipment and techniques which are not always available. Thus, in this research we decided to use macroscopic images only. The macroscopic characteristics of wood are those that can be seen with the naked eye or with the help of a small magnification (there is no general agreement on the level of magnification that can be considered “small”). In most cases, the set of macroscopic characteristics unequivocally defines each species, which allows its identification (Díaz-Vaz, 1979). Traditionally, identification at a macroscopic level is a manual process that requires a high degree of knowledge to observe and differentiate certain anatomical structures present in a wood sample. Observations are performed on each of the three cutting planes: transverse (cross) section, radial section and tangential section (see Figure 1), with the help of a hand lens (Wiedenhoeft, 2011a,b). Then, by using an identification key, wood species atlases or field guides and manuals, the expert determines the species of the wood sample. Therefore, the accuracy of such identification critically depends on the observer's expertise.

Wood species identification is a very complex task even for experts. For this reason it has been addressed from a computational view point. The most recent approaches are based on deep learning. This is motivated by the success achieved with these techniques in automatically identifying plant species based on pictures of leaves, flowers and other plant components different from wood cuts (Carranza-Rojas et al., 2017; Goëau et al., 2017). The success achieved from this perspective has been remarkable, for example, the Pl@ntNet application^1^ is capable of identifying thousands of plant species of the world's flora from an image. However, it has already been documented that image datasets of wood cuts are few, uneven across taxa, and/or small; consequently, not ideal for deep learning approaches (Figueroa-Mata et al., 2018a). Perhaps, the main reason is that the process of acquisition of samples and both macroscopic and microscopic images of wood is complex and expensive. Besides human experts do not require, for example, many wood samples of the same species to make an identification. Nevertheless, the availability of smartphones equipped with high-quality cameras and low-cost digital microscopes that take photographs comparable to microscopic images is making feasible a change.

Below is an overview of the most recent publications in this field. Kwon et al. (2017) proposed six different CNN models (variants of LeNet and MiniVGGNet architetures) to identify five softwood species from Korea. A smartphone camera was used for obtaining macroscopic wood images. The best accuracy achieved was 99.3% with a LeNet3 architecture.

Ravindran et al. (2018) proposed one variant of the VGG16 model pre-trained on ImageNet to identify 10 neotropical wood species of the Meliaceae family. They tested the proposed model to species and genus levels, achieving an accuracy of 87.4 and 97.5% respectively.

In Apolinario et al. (2018) a small CNN architecture was proposed to identify seven commercial timber species from Peru. From each image, they extracted patches of three different sizes, namely, 32 × 32, 64 × 53 and 128 × 128 pixels, with which they built three datasets for their experiments. A portable digital microscope connected to a personal computer was used to get the wood images. The best accuracy achieved was 94.05% for the 128 × 128 pixels image dataset.

Oktaria et al. (2019) conducted a comparison of four CNN architectures: Kayu30Net, AlextNet, ResNet and GoogNet on a datset composed of 30 species of wood images which were obtained from the Xylarium Bogoriense, Indonesia. However, they did not specify further details about the database or the training process of the models, e.g., if they use some kind of transfer learning.

Ravindran et al. (2019) applied the ResNet34 model pre-trained on ImageNet to identify 38 wood species corresponding to 15 genus of commercial interest in Ghana. They reported an accuracy of 97.0% in the laboratory and 72.0% in field testing.

In Apolinario et al. (2019) the authors proposed a CNN architecture based on Inverted Residual Mottenecks Blocks (IRB) and depthwise convolutional layers that allow the identification of known species and the clustering of those that are not. In this sense, the proposed architecture is a classifier and clustering algorithm. For their experiments they used two databases, the first one composed of images of 16 wood species from Perú and the second one is the Forest Species Database Macroscopic (FSD-M) ^2^ composed of images of 41 wood species from Brazil (Filho et al., 2014). Accuracies achieved were higher than 91% for seen and unseen species during the training phase.

Yang et al. (2019) applied transfer learning to the pre-trained VGG19 model on ImageNet dataset. Then, they used this already tuned model for the classification of 25 wood species, reaching an average accuracy per species of 93.63%. They also applied this strategy to the VGG16 and InceptionV3 models, reaching an accuracy of 92.72 and 92.41% respectively.

Yusof et al. (2020) reported 100% accuracy when applying transfer learning to the ResNet50 model. The database they used was composed of 20 tropical wood species.

In Verly Lopes et al. (2020), an InceptionV4-ResNetV2 model pre-trained on ImageNet was used to classify 10 North American hardwoods species. The accuracy achieved was 92.6%.

Summarizing, most of the papers published about tree species identification from wood images share the following aspects:

- Use macroscopic wood images, specifically from cross-sectional woodcuts;
- apply transfer learning techniques to train the models proposed;
- the number of species considered is small, not greater than 40;
- do not indicate if their training/testing processes avoid the Same-Specimen-Picture Bias (SSPB), which substantially, but erroneously, increases accuracy, as was reported by Carranza-Rojas et al. (2018);
- have not developed mobile applications capable of accurately and quickly identifying wood samples to prevent illegal logging and mislabeling.

The main goal of this paper is to describe how we have applied deep learning techniques to the identification of Costa Rican native wood species. More specifically, we address the following three problems:

- Develop a new protocol that is practical, non-destructive, uses less space in xylotheques, and more quickly results in a number of samples appropriate for deep learning applications.
- Implement a CNN for automated tree species identification based on macroscopic images of wood cuts that has an average top-1 accuracy higher that 70%, for at least 70 tree species, while avoiding experimental biases such as the SSPB (Same-Specimen -Picture Bias) described in Carranza-Rojas et al. (2018).
- Develop a mobile application that uses the CNN as back-end to identify Costa Rican native tree species from images of wood cuts.

## 2. Materials and Methods

One of the problems researchers face when they attempt to apply deep learning techniques to wood species identification is the lack of macroscopic image databases. To our knowledge, the only open-access database is the Forest Species Database — Macroscopic (FSD-M) of the Laboratório Visão Robótica e Imagem (Filho et al., 2014). It currently comprises 2,942 macroscopic images from 41 different forest species of the Brazilian flora. Because we wanted to test our research on species from Costa Rica and with a larger dataset, we collected wood samples that grow in Costa Rica and integrated them to the Víctor Rojas Xylotheque of the Costa Rica Institute of Technology.

To collect and process the wood samples, as well as to create the image database, we developed new protocols that standardize the involved processes. The following two subsections briefly describe the protocols created and first mentioned in Mata-Montero et al. (2018).

### 2.1. Sample Collection Protocol

**Figure 2:**
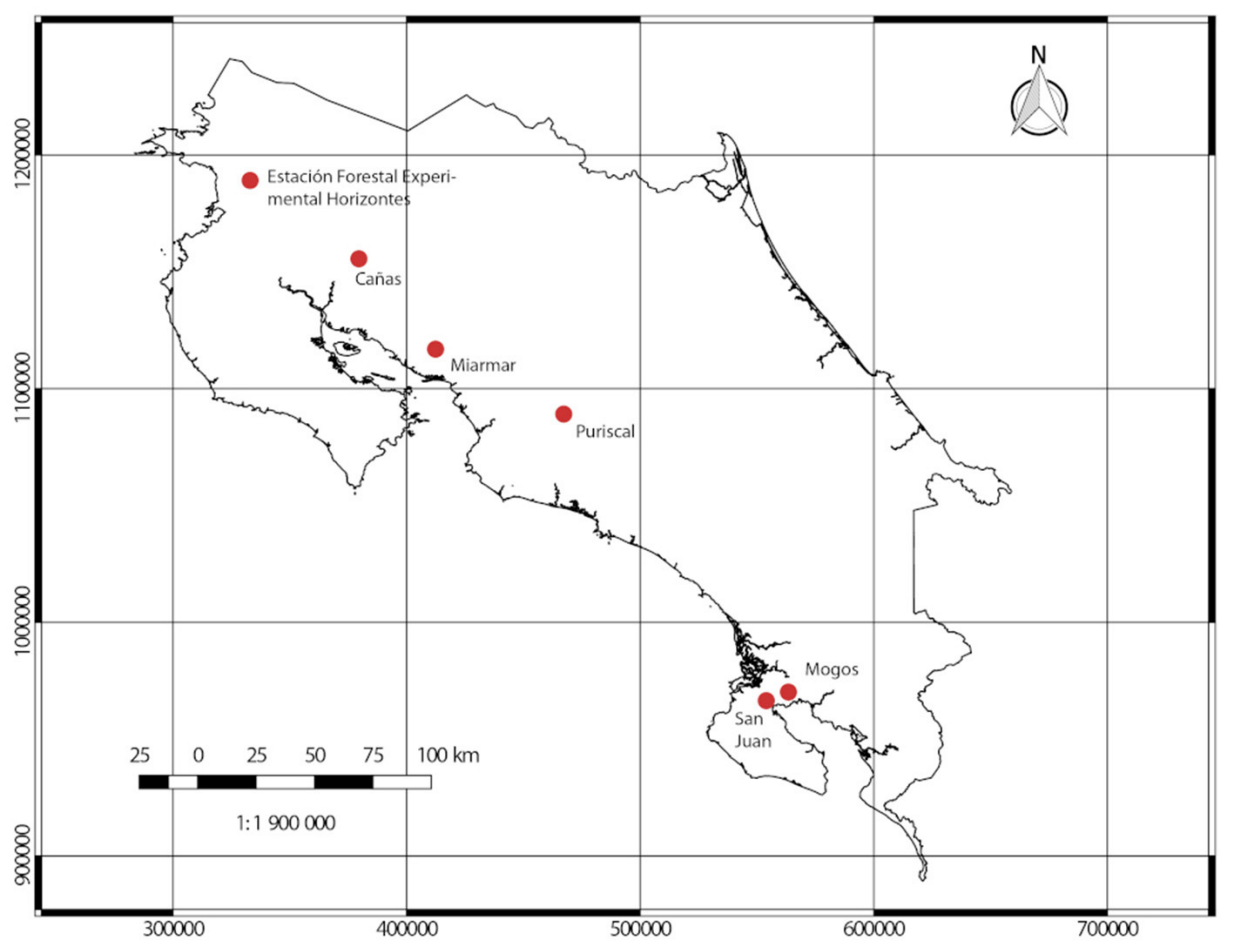
Location of forest reserves selected for sample collections.

All wood samples were collected from forests located along the Pacific Coast of Costa Rica (see Figure 2); specifically, collections were made at six pre-established sites:

1. Estación Experimental Horizontes, located in Guanacaste (10°42′10″*N*, 85°33′12″*W*) at an altitude of 120 m.
2. Cañas, located in Guanacaste (10° 27′ 02″ N, 85° 06′ 22″ W) at an altitude of 100 m.
3. Miramar forests, located in Puntarenas (10°01′29″*N*, 84°14′04″*W*) at an altitude of 270 m.
4. Parque Nacional La Cangreja, located in Puriscal (9.69°*N*, 84.36°*W*) at an altitude of 800 m..
5. Mogos forest, located in Península de Osa (8°45′00″*N*,83°22′59″*W*) at an altitude of 40 m.
6. San Juan, located in Península de Osa (8°39′02″*N*, 83°27′53″*W*) at an altitude of 35 m.

In this research we do not use the information about altitude because the number of samples is still relatively small. However, this information is very important for other applications and could be used for machine learning applications once a larger dataset is created. If the xylotheque where samples are maintained has access to a GIS system and altitudinal maps, then altitude is generated by the GIS, otherwise it is critical to record it for other uses.

**Figure 3:**
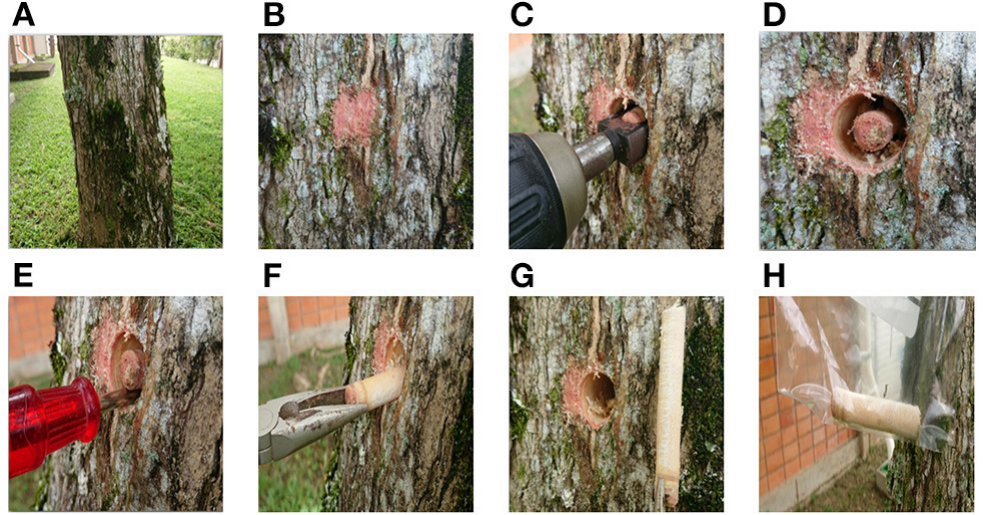
**(A–H)** Extraction process of a sample of wood.

The protocol developed for the extraction of samples in the field includes the following steps:

1. **Selection and evaluation of tree specimen**. The tree specimen must be a healthy tree with a diameter at breast height (DBH) greater than or equal to 20 cm. The distance between the selected trees must be at least 15 m., in order to increase the variability between species. Once the tree is selected, its features are recorded: species, diameter, number of tree specimen, location, etc. Finally, a picture of the tree is taken from 1 m. distance (Figure 3A).
2. **Selection and cleaning of point of perforation**. A place is selected on the tree, free of branches and deformities. A 5 *cm*^2^ spot is cleaned, 1 m. above ground level, and bark is removed (Figure 3B).
3. **Perforation of trunk**. A perforation is done by using a battery operated 20v drill. The drill is equipped with a 1/2” diameter plug cutter and the perforation depth is approximately 80 mm. The perforation process is non-destructive. Healing of the tree takes 6–10 months. Once the perforation is finished, the drill is removed and the sample is exposed (Figures 3C,D).
4. **Extraction of the sample**. A pricker is introduced to break the basis of the cylinder and the sample is removed with needle-nose pliers (Figures 3E–G). Each sample has a length of approximately 75 mm and a 12.5 mm diameter.
5. **Sample storage and preservation**. The sample is stored in a sealed plastic bag that contains a 10 ml solution of 8 ml of water and 2 ml 95% alcohol. The sample is now ready to be taken to the laboratory (Figure 3H).

### 2.2. Sample Processing Protocol

**Figure 4:**
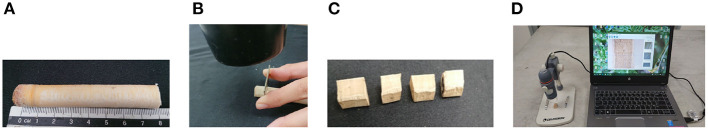
**(A–D)** Sample processing.

Once a sample has been extracted it is processed in the laboratory to prepare it for photography. This protocol includes the following steps:

1. **Cleaning the sample**. In the laboratory, each wood sample is cleaned in order to eliminate traces of bark and cambium, leaving only sapwood and heartwood. The sample is measured and weighed (Figure 4A).
2. **Sectioning the sample**. Depending on the actual length of each wood sample, each one is turned into four or five smaller cylindrical sections of 13 mm length by using a precision blade (Figure 4B).
3. **Cutting the sections into cubes**. Each face of each smaller cylindrical section is turned into a cube by using a tungsten blade (Figure 4C). The approximate size of each cube is 10 *mm*^3^.
4. **Taking pictures**. Each cube is photographed twice, first in fresh/green condition, then they are subjected to a drying process during 72 h at 65°*C*, and again are photographed in dry condition (Figure 4D). For each cube, three pictures are taken: one for a cross section, one for a tangential, and one for a radial section. Photographs are taken with a 20X magnification Celestron^©^ 5 Megapixel electronic stereoscope.
5. **Building up the database**. Finally, metadata such as date and place of extraction, specimen ID, dimensions, and weight, among others, are recorded for each cube (Mata-Montero et al., 2018). Additionally, for each picture, the picture itself, the type of cut (cross, tangential or radial), cube it belongs to, and stage of the wood when the picture was taken (fresh, dry) is recorded and stored.

### 2.3. Dataset

**Figure 5:**
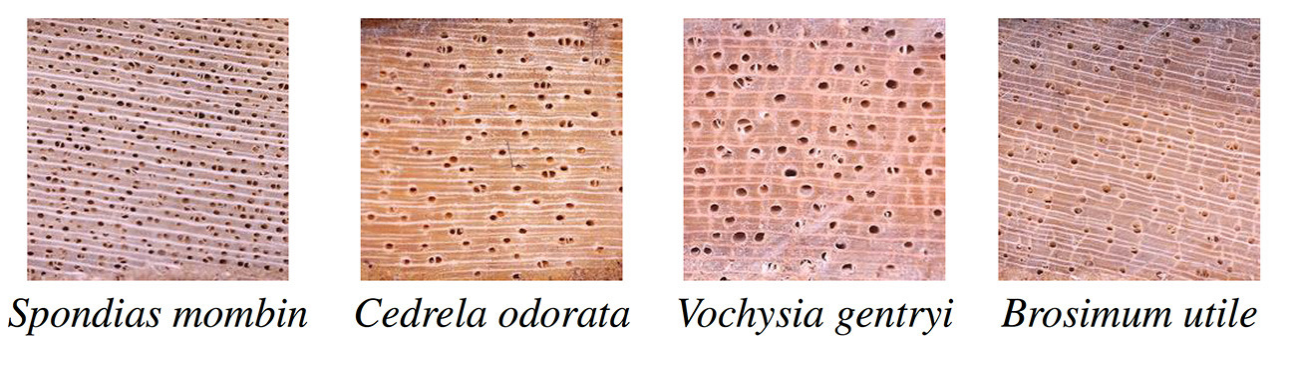
Some of the images in the database.

The complete image database consists of pictures of 656 samples from 147 tree species from Costa Rica. It includes 42 families and 110 genera. It comprises about 3,516 images of each of the three sections, namely, transverse, radial and tangential, in two conditions, dry and fresh; that is, approximately, 3, 516 × 3 × 2 = 21, 096 images. Each image is in uncompressed JPG format with 2, 592 × 1, 944 pixel resolution, although there are some few images of smaller resolution. Appendix 5A lists the scientific names of each of the 147 species. Figure 5 shows some of the images in the database. Because this database is highly imbalanced, with several species having only one specimen, a subset with 75 species, described in Subsection 2.5.2, was used in the experiments.

### 2.4. Hardware and Software Used

All experiments were conducted on a desktop computer with an Nvidia TITAN RTX GPU with 24GB GDDR5 of memory and a AMD Ryzen 9 3900X 12-Core Processor with 32 GB of memory. As to software, Tensorflow version 2.2.0 and Keras version 2.3.0-tf were used to develop the CNN.

Photographs are taken with a 20X magnification Celestron^©^ Labs S20 Stereo Microscope. Figma 6.0 was used for the graphic design and Ionic Framework 4.0 for the development of the mobile application Cocobolo. Node.js 8 (version 12.16.1) was used as an intermediary for user management, database queries and communication between the front and back-end. The creation and management of text and image databases was implemented in MongoDB 4.4. Finally, we used TensorFlow Serving (version 2.0) to deploy the CNN model for production. The back-end runs under Ubuntu 18.04 and was developed using TensorFlow (version 2.2) and the Keras module.

### 2.5. Convolutional Neural Network Architecture

Since the number of images was small to train a convolutional model from scratch, we applied the fine-tuning technique using VGG16 as a base model, pre-trained with the ImageNet data set.

For fine-tuning purposes, we removed the softmax layer of the base model and replaced it with our own, adding a Global Average Pooling layer, two dropout layers and two dense layers. In addition, we froze the weights for the first 10 layers so that they remained intact throughout the fine-tuning process.

We also experimented with other base models such as ResNet50 and MobileNet, both ImageNet pre-trained, but obtained similar results. For example, with ResNet50 the Voting Rule top-1 accuracy was 49.3%, compared to 70,5% when using VGG16, as described in the Results Section.

#### 2.5.1. Data Augmentation

Deep learning models often implement a data augmentation stage to reduce overfitting and improve performance in imbalanced class problems (Goodfellow et al., 2016; Wong et al., 2016). Because the number of images per species is small for deep learning approaches, we applied two different data augmentation techniques.

**Figure 6:**
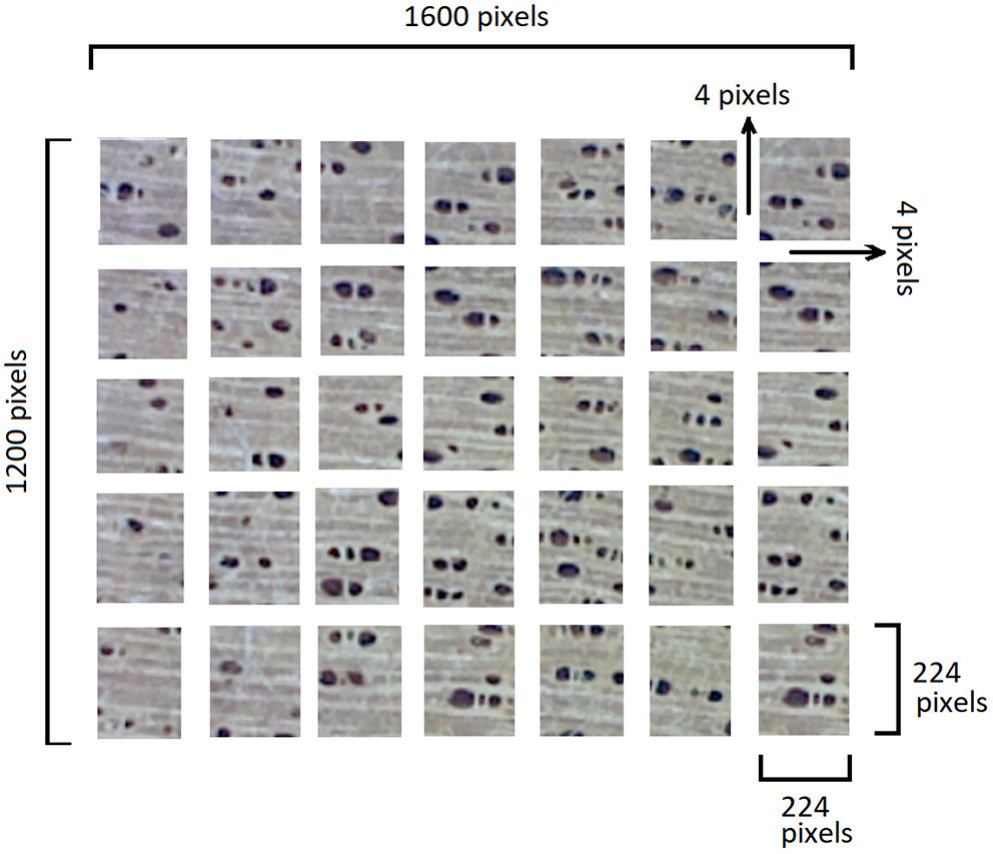
Dividing the original image into sub-images (taken from Figueroa-Mata et al., 2018b).

First, we divided each original image into several non-overlapping sub-images (patches) that are four pixels apart. For instance, if the resolution of the original image is 1, 600 × 1, 200 pixels, we can obtain 35 sub-images size 224 × 224 pixels. Figure 6 illustrates this technique. We used an input size of 224 × 224 pixels due to physical limitations of the hardware used, specifically the graphics card memory. When there are memory limitations, at least two variables must be balanced: batch size and image dimension. It is recommended to use a relatively large batch size to generate good approximations of the gradient of the loss function. Furthermore, the images must also be large enough to capture a goog number of discriminating features. There is clearly a trade-off between these two variables. We consider 224 × 224 images and batches of 32 images to be a reasonable compromise since smaller images do not capture the multiple anatomical features present and larger images would force us to use smaller batch sizes.

Secondly, we implemented, on the fly, transformations such as rotation (value = 30), horizontal flip (value = True), vertical flip (value=True), width shift (value = 0.2), height shift (value = 0.2) and zoom (value = 0.3). For this, we used the **ImageDataGenerator** class of **Keras**, with the values indicated.

#### 2.5.2. Training and Validation Datasets

Since the number of specimens per species in the database varies from 1 to 19 (see Table 1), we decided to diminish the imbalance by selecting those species with at least 5 specimens. As a result, we obtained 75 species of which, for example, 40 have 5 specimens. Appendix 5A shows, highlighted in bold, the scientific name of the selected species.

**Table 1:** Number of species and specimens in dataset.

**# species**	**23**	**18**	**16**	**12**	**41**	**10**	**2**	**8**	**3**	**4**	**4**	**1**	**1**	**1**
# specimens per species	1	2	3	4	5	6	7	8	9	10	12	14	15	19

All datasets used in the work described in the Introduction Section use cross section images exclusively. This is possibly because cross sections tend to contain more information than radial and tangential cuts. Additionally, in Figueroa-Mata et al. (2018), it was confirmed that cross sections are more significant when training a convolutional neural network of 40 species from Costa Rica. Those 40 species are a proper subset of the set of species in CRTreeCuts. We are not aware of any other study that compares the relative significance of wood cuts for CNN-based tree species classifiers. Based on these facts we decided to use only cross sections — in dry condition, as this is their condition in xylotheques — to train, validate and test our models, leaving for future research the (combined) use of other cuts.

Images of the these 75 species were divided as follows: 70% for training, 20% for validation, and 10% for testing, resulting, after avoiding the Same-Specimen-Picture Bias (SSPB) (Carranza-Rojas et al., 2018) in approximately 1,712 images for training, 522 for validation and 426 for testing; and finally, after applying the data augmentation techniques described in section 2.5.1, in approximately 95,446 for training, 29,109 for validation and 23,187 for testing. We say that SSPB is avoided (it is absent) if all the images of the dataset are distributed so that, for each sample (specimen) *S*, its images are used exclusively in one of the following sets: training, validation or testing. Avoiding SSPB is very important, otherwise, it could lead to fictitiously good results as has been documented in Carranza-Rojas et al. (2018) and our own experiments.

## 3. Results

### 3.1. Accuracy of CNN

We conducted several experiments with the convolutional model described in Section 2.5. Different values for hyper-parameters such as learning rate and batch size were tested, as well as optimizers such as SGD, Adadelta, Adam, and RMSprop.

The following two definitions of average top-k accuracy were used. The first one applies to image patches (subdivisions) obtained after data augmentation. The second definition applies to complete images.

Let *T* be the set of images *I* used for testing after data augmentation is applied. We define Accuracy_*k*_, the average top-k accuracy achieved by the model with set *T* as follows:


(1) $$\begin{array}{r} {\text{Accuracy}}_{k}=\frac{1}{\left|T\right|}\underset{I\in T}{\sum }\text{hit}\left(k,I\right) \end{array}$$

where hit(*k, I*) is a boolean function that indicates if one of the top-k candidate species in the ranking generated by the model is a correct identification of image *I*. An analogous definition can be given for the average accuracy achieved at the species level.

The average top-k accuracy can also be defined by a voting rule (VR) similar to the one introduced by Siew et al. (2017). More formally, we define VRAccuracy_*k*_, the average top-k accuracy with VR, as


(2) $$\begin{array}{r} {\text{VRAccuracy}}_{k}=\frac{1}{\left|C\right|}\underset{I^{\prime }\in C}{\sum }\text{VRhit}\left(k,I^{\prime }\right) \end{array}$$

where *C* is the set of complete (not subdivided in patches) images used for testing and VRhit(*k, I*′) is a boolean function that is true iff the correct species is in the set of *k* candidate species that more often are predicted correctly when all patches *p* of image *I*′ are tested with hit(*k, p*). This second measure of accuracy is more realistic, as it is associated with a complete image but Accuracy_*k*_ is useful for training and validation, which is the approach we used.

**Figure 7:**
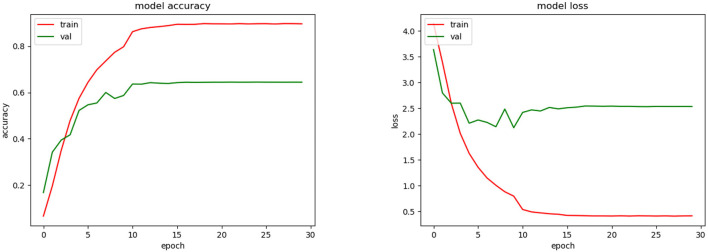
Training accuracy and loss for CNN model.

The best average top-1 accuracy achieved for validation during the training phase was 65.6% (see Figure 7). For this, we used the SGD optimizer with a learning rate automatically fitted according to the change of the validation accuracy. Initially, the learning rate was 0.01 and was fitted by a factor of 0.1. After 10 epochs the validation accuracy did not improve significantly. In the testing phase, the average top-1 and top-3 accuracies were 59.9 and 76.5%, respectively.

Additionally, we calculated the top-1 and top-3 average accuracies using VR and equation (2). With VR, we achieved a 70.5% top-1 average accuracy and 80.3% top-3 average accuracy (see Appendix 5B).

Appendix 5B also presents the MRR for each species as well as the average over all 75 species. The mean reciprocal rank (MRR) was computed with the following formula:


(3) $$\begin{array}{r} MRR=\frac{1}{\left|Q\right|}\underset{i\in Q}{\sum }\frac{1}{rank_{i}}, \end{array}$$

where *Q* is the set of testing images and *rank*_*i*_ is the ranking position achieved by the *i*-th image. *Q* consists of patches (Figure 6) generated in the data augmentation process, i.e., no voting rule is used.

Of the 75 tested species, there were six that the CNN could not identify even once, that is, the average top-1 and top-3 accuracies were both 0, when using the voting rule. The scientific name of these species and the number of specimens per species are shown in Table 2.

**Table 2:** Species never correctly identified by the CNN.

**Scientific name**	**# Specimens**
*Pouteria filipes*	5
*Brosimum lactescens*	5
*Garcinia madruno*	5
*Tapiria guianensis*	9
*Drypetes brownii*	5
*Pachira aquatica*	5

**Figure 8:**
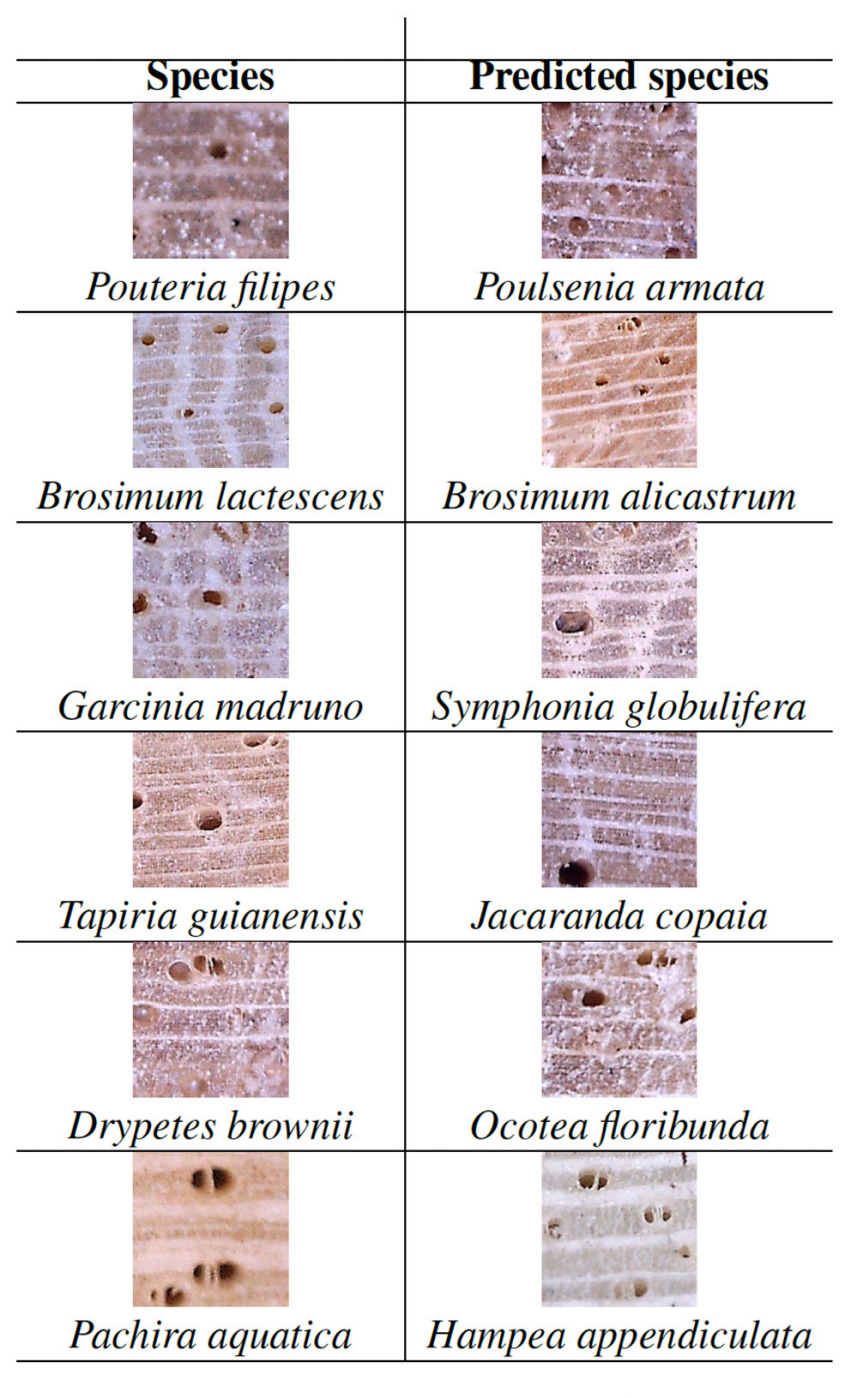
Images of species never correctly identified and the top-1 predicted species.

To clarify what could be happening we analyzed in detail the classification that the CNN carried out for these species. Figure 8 shows an image of each of these six species and the image of the predicted species. It is interesting to highlite that, for each species, the top-1 (incorrectly) predicted species was always the same. For instance, the network always confused the species *Pouteria filipes* with the species *Poulsenia armata*, which seems reasonable, since both species images are very similar, as we can see in Figure 8. Besides, there are some close taxonomic relationships for three of the six pairs of species in Table 2 that may help partially explain the failures (even though there are other pairs of species that are also related but predictions are much better). Specifically, three pairs of species belong to the same family, and one of them also belongs to the same genus. Species *Garcinia madruno* and *Symphonia globulifera* both belong to the Clusiaceae family, while *Pachira aquatica* and *Hampea appendiculata* belong to the Malvaceae family. Finally, *Brosimum lactescens* and *Brosimum alicastrum* belong to the Moraceae family and have the same genus.

Additionally, Table 2 shows that the number of specimens of each of these six species is always 5, except for *Tapiria guianensis*, which has 9 specimens. However, there are species such as *Cordia alliodora, Hymenaea courbaril, Bursera simaruba, Trichilia pleeana*, and *Lonchocarpus macrophyllum*, among others (see Appendix 5B), for which having only 5 specimens is not problem. They are all classified with over 85% top-1 accuracy.

Another interesting result is that the misclassification performed by the CNN for species in Table 2 is one-way, i.e., the network always confuses the species in the first column with the species in the second column (Figure 8), but all the species in the second column are identified with high accuracy, namely, 100% top-3 accuracy and more than 75% top-1 accuracy (see Appendix 5B). This suggests that the number of specimens is not enough for the network to learn how to differentiate species correctly and, therefore, that future collecting efforts should try to focus on those species in order to improve the overall identification accuracy.

**Figure 9:**
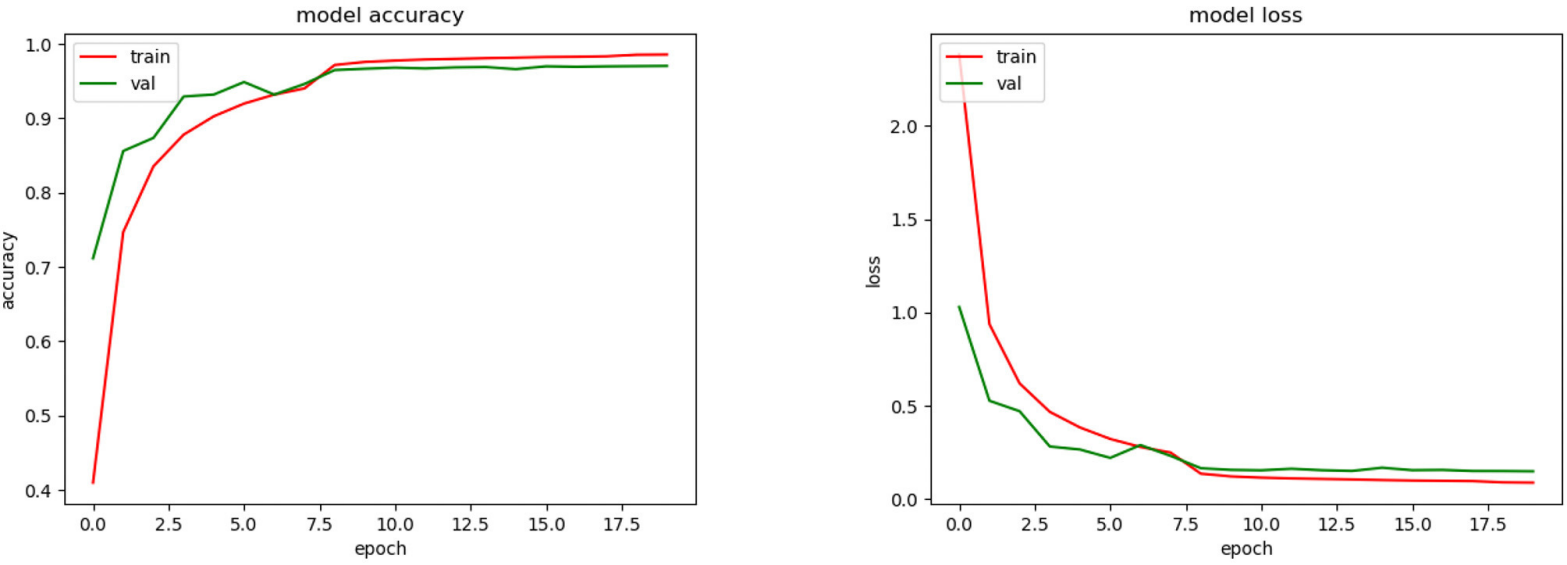
Training accuracy and loss for CNN model with SSPB bias.

As we could see during the training phase (see Figure 7), overfitting was present. This was caused by the restrictions imposed to avoid the SSPB bias (Section 2.5.2). As was mentioned by Carranza-Rojas et al. (2018), if the bias is present in the training, validation and testing phases, the accuracies are fictitiously increased. To highlight this fact, we conducted an experiment in which SSPB was present. Figure 9 shows the top-1 accuracy and loss functions. As we can see, the top-1 accuracy achieved was 97.5% for validation, which is much better than the 65.5% achieved when we avoided the SSPB bias (Figure 7). Once the model was trained we evaluated them on the testing set and achieved a top-1 accuracy of 97.3%, which is also considerably better than the top-1 accuracy achieved in the testing phase if SSPB is avoided. Carranza-Rojas et al. (2018) report differences of around 10%, whereas in this case the differences are larger than 25%. We believe this is because different pictures of one specimen are very similar, as they were all part of the same cylinder that was removed from the tree. Thus, SSPB should definitely be avoided in the development of deep learning tools for the identification of tree species based on wood cut images.

**Figure 10:**
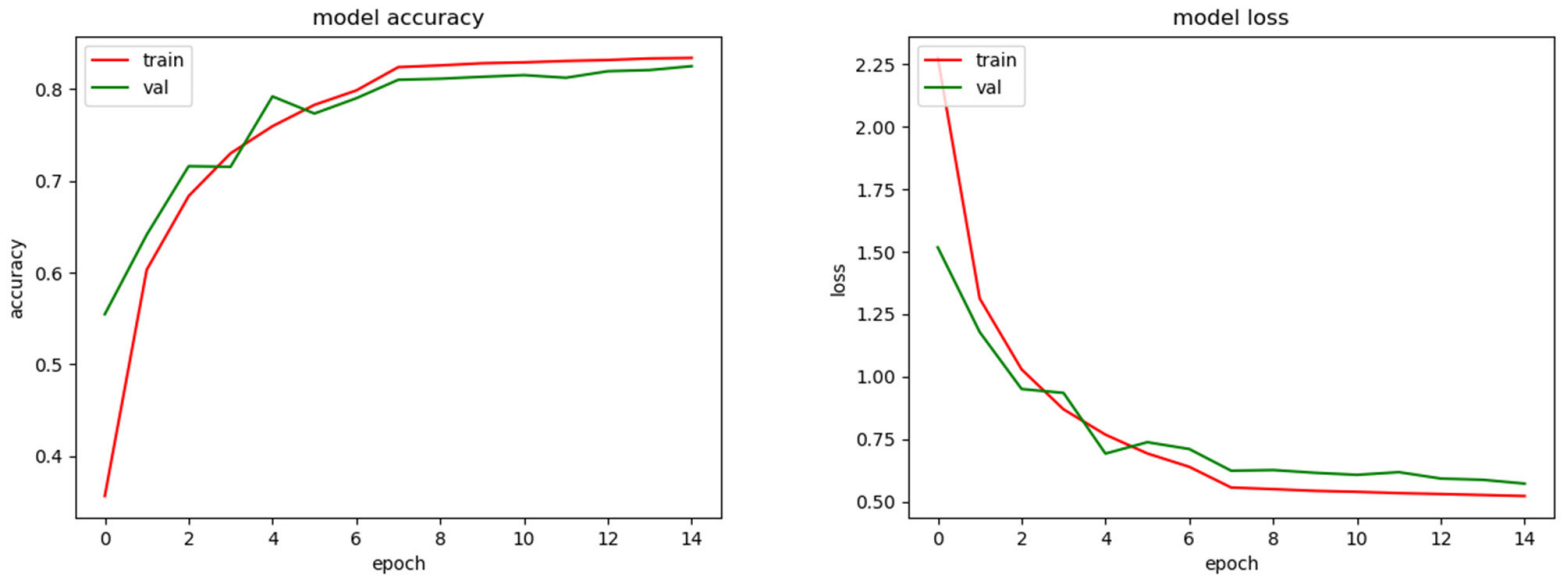
Training accuracy and loss for CNN model with FSD-M dataset and (possibly) SSPB bias.

In order to test the reliability of the model, it was also trained and tested on a different dataset. For this purpose, we used a dataset of images of species from Brazil (FSD-M) (Filho et al., 2014). Figure 10, shows the accuracy and loss obtained during the training phase of the model. Additionally, once the model was trained, we tested its performance on a subset of images that was not used in the training phase and obtained an average 83.8% top-1 and 95.2% top-3 accuracy.

As an additional result of this work, preliminary tests show that the samples collected with the proposed methodology can be used not only to develop deep learning applications such as Cocobolo, but also for the anatomical characterization of Costa Rican timber species (Valverde et al., 2020). This means that samples obtained with the proposed protocol can be used in xylotheques for other applications just like the larger samples they traditionally collect and hold.

### 3.2. Cocobolo Mobile Application

An application capable of identifying tree species from a wood image can be of great value in legal, commercial, industrial and forensic contexts, because it can support legal experts, forestry inspectors and customs officials to do field identifications in a simple, fast and accurate way. For example, forestry inspectors could determine if a wooden cargo is properly labeled to ensure compliance with the established regulations.

As a supplement to the convolutional neural network designed for the identification of tree species from Costa Rica, we developed a mobile application for smartphones compatible with the Android operating system. We decided to call the mobile application Cocobolo (*Dalbergia retusa*), because it is an endangered native tree species of Central America whose wood is beautiful and of great commercial value.

Unlike mobile tools for plant identification based on flowers, leaves, and other plant components, Cocobolo is a tool developed for a specialized audience that includes xylotheque users, law-enforcement officials, and tree species taxonomists, among others. Its work flow starts with a set of wood samples that have been already collected, treated, and need to be photographed and identified. Thus, in general, users of Cocobolo are not expected to use it to carry out identifications in the field (unless they take along the specialized equipment described in Sections 2.1 and 2.2 or have access to samples already collected). Most of its functionality can be achieved with a website that uses the developed back-end to do the identifications. We chose to develop a mobile application before a website (currently underway) to facilitate image capturing, to provide a personalized environment to manage pictures and their identifications, and to refine use cases for future versions of both, mobile applications and a website.

Because its back-end has been thoroughly trained and tested, the testing phase of the back-end is a simulation of the expected accuracy of Cocobolo with collected wood samples. All photographs used were taken with a 20X magnification Celestron©, 5 Megapixel electronic stereoscope. However, smartphone cameras are already getting close to that level of magnification and will have that feature in the near future. For the time being, we recommend the use of a clip-type lens. They are very inexpensive and meet the 20X magnification requirement.

Cocobolo has of two components. First, a front-end, which is the interface that captures queries, takes pictures, and presents the results on the smartphone. Secondly, the CNN that acts as the remote back-end, performing the identification. Cocobolo was designed following the methodology proposed by Hernández-Castro for the development of software tools (Hernández-Castro, 2016). The software tools employed are described in Section 2.4.

Cocobolo allows users to identify pictures of wood stored in the phone's gallery or use the camera to take a picture and identify it. The identification process responds with the three most probable species, their scientific name, their common name, an estimated level of accuracy, and one image for each species. Users can also consult general information about the species with which the convolutional neural network was trained and share their identification by email or using WhatsApp.

**Figure 11:**
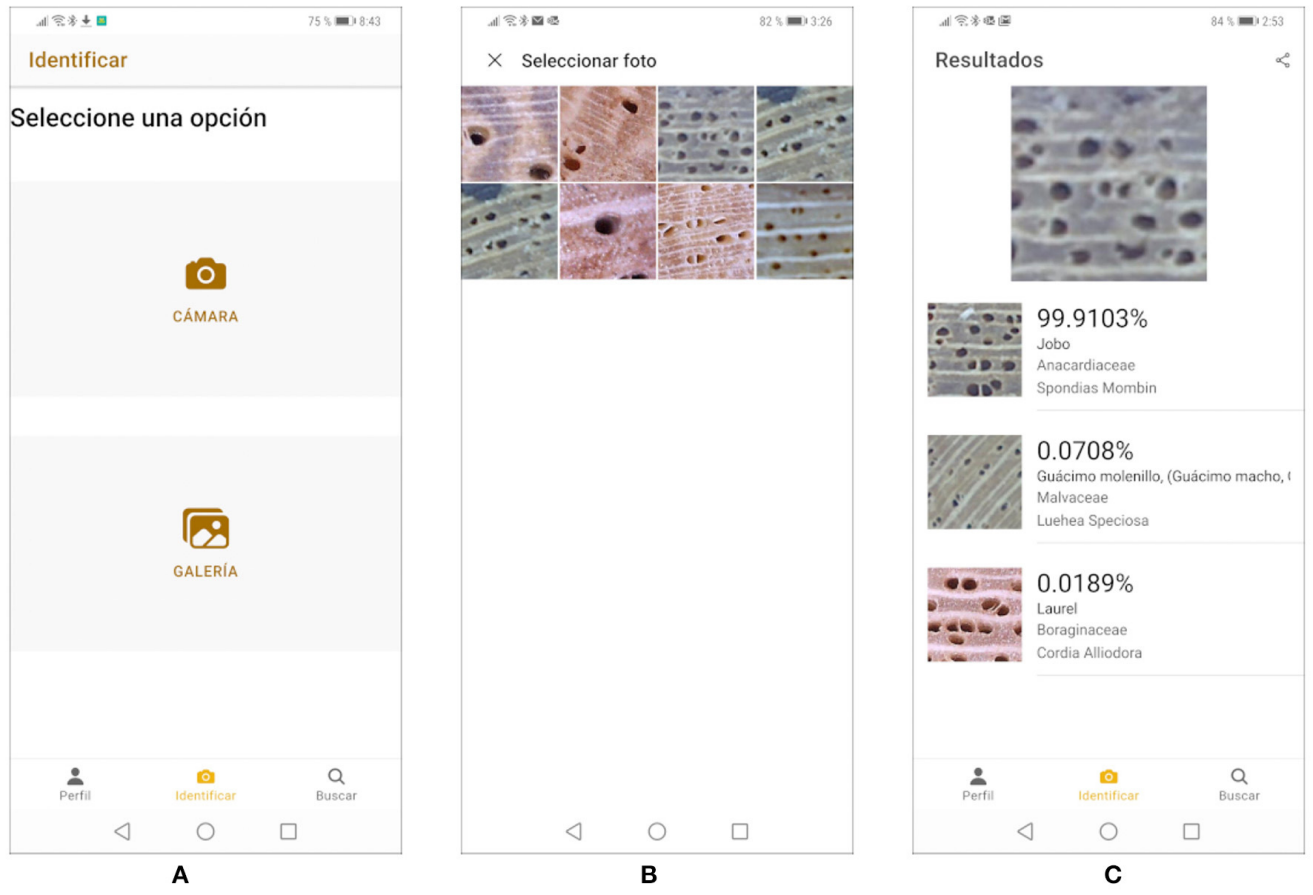
**(A–C)** Cocobolo mobil application.

Figure 11 shows on common use case. When the user selects the option “Identify,” the screen in Figure 11A is displayed and the user can choose between “Gallery” or “Camera”. The “Camera” option activates the smartphone's camera for the user to take a new picture. The “Gallery” option shows the smartphone's photo gallery which, in this case, contains 8 previously stored photos, as shown in Figure 11B. Once the picture is selected, it is identified after clicking the button “Identify.” For instance, if the user chooses the third photo in the top row in Figure 11B, Cocobolo responds with the ranking shown in Figure 11C. Cocobolo has been successfully tested with up to five concurrent users and is being fine-tuned to improve its performance before public deployment.

## 4. Discussion

An alternative solution to the traditional way of identifying tree species from macroscopic images of wood cuts was presented. The approach has been to build a CNN by applying the fine-tuning technique to a base VGG16 model pre-trained with ImageNet. The resulting model was then trained with a new database composed of cross-sectional wood images of 75 Costa Rican tree species. The achieved top-1 and top-3 accuracies during the testing phase were 76.5 and 80.3%, respectively. We consider this very good, given that this is the first attempt with this dataset and that the number of species is relatively large compared to previous work in this domain. Consequently, this CNN was used as back-end for a mobile application named Cocobolo. This mobile application allows the identification pictures of wood stored in the smartphone's gallery or use the camera to take a picture and identify it. Cocobolo responds with the 3 most probable species, their scientific names, their common names and one image for each species. In addition, it is possible to share the identification results by email or WhatsApp. Cocobolo is currently undergoing fine-tunning for efficient integration of its components with larger number of concurrent users.

The methodology used to conduct the training, validation and testing sets avoided the well-known Same-Specimen-Picture Bias. The presence of this bias is often overlooked in similar research. However, in this domain we experimentally confirmed that it would have erroneously increased the average accuracy by more than 25%. Therefore, future work should consider and avoid this type of bias. Consequently, image databases of wood cuts must be documented with enough data to avoid potential biases. Specifically, programmers should be able to always test, for each pair of images {*I*_1_, *I*_2_}, if specimenID(*I*_1_) = specimenID(*I*_2_).

We proposed an innovative workflow that defines protocols for—non-destructively—collecting samples in the field, processing the samples, taking photographs, and annotating these pictures in a database. As a result, we have supplemented the Costa Rica Institute of Technology xylotheque with 656 wood samples of 147 tree species from Costa Rica. In addition, a database with 21.096 images was created.

The identification of tree species is critical to support their conservation, sustainable management, and, mostly, to fight illegal logging. However, an immediate challenge to make this approach more effective is to build larger datasets with more species and more specimens per species. In this respect, we are already using the same protocols to enhance *CRTreeCuts* so that it comprises at least 200 tree species and at least 10 specimens per species. This corresponds to approximately 8.5% of the number of tree species in the country and 15% of timber species. However, even though it is critical to increase the size and taxonomic coverage of datasets, field trips to collect wood samples and lab work on those samples is still slow and costly. Thus, recent approaches to deep learning with small datasets should be explored for this domain, among them, zero-shot (Larochelle et al., 2008), one-shot (Li Fei-Fei et al., 2006; Koch et al., 2015; Vinyals et al., 2016), few-shot or *k*-shot learning (Chen et al., 2019; Wang et al., 2020), and Siamese networks (Baldi and Chauvin, 1993; Bromley et al., 1993; Figueroa-Mata and Mata-Montero, 2020).

## Data Availability Statement

The original contributions presented in the study are included in the article/supplementary materials, further inquiries can be directed to the corresponding author.

## Author Contributions

EM-M, DA-A, GF-M, and JC: project design. GF-M and EM-M: drafting and refining the manuscript and experiment design. GF-M: experiment programming and refinement. GF-M, EM-M, and JC: definition of Cocobolo use cases. GF-M: testing of Cocobolo versions. JC: design and testing of sample collection protocol, coordination of sampling, sample processing, and digitization. JC and NZ-V: collection of wood samples in the field. All of the authors have read and approved the manuscript.

## Funding

We acknowledge and thank the financial support from the Vicerretoría de Investigación, Dirección de Posgrados and Programa de Doctorado en Ingeniería of Costa Rica Institute of Technology.

## Conflict of Interest

The authors declare that the research was conducted in the absence of any commercial or financial relationships that could be construed as a potential conflict of interest.

## Publisher's Note

All claims expressed in this article are solely those of the authors and do not necessarily represent those of their affiliated organizations, or those of the publisher, the editors and the reviewers. Any product that may be evaluated in this article, or claim that may be made by its manufacturer, is not guaranteed or endorsed by the publisher.

## Footnotes

^1^ https://plantnet.org/

^2^ https://web.inf.ufpr.br/vri/databases/forest-species-database-macroscopic/
